# Color Stability of Pediatric Restorative Material Over Pediatric Drug Formulation

**DOI:** 10.7759/cureus.42953

**Published:** 2023-08-04

**Authors:** Anant Singh, Chhavi Grover, Dwij Raina, Aanchal Pandey, Akondy Sri Chaitanya Krishna

**Affiliations:** 1 Pedodontics and Preventive Dentistry, Kalka Dental College, Meerut, IND; 2 Orthodontics and Dentofacial Orthopaedics, ITS Dental College, Ghaziabad, IND; 3 Dentistry, ITS Dental College, Ghaziabad, IND; 4 Pedodontics and Preventive Dentistry, Sardar Patel Post Graduate Institute of Dental & Medical Sciences, Lucknow, IND; 5 Epidemiology, University of Iowa, Iowa City, USA

**Keywords:** glass ionomer cement, composite resin, pediatric drug, dental restoration, colour stability

## Abstract

Aim: To investigate the color stability of light-cured (LC) restorative material in different pediatric drug formulations.

Method: Two distinct restorative materials, specifically LC resin and LC glass ionomer cement (GIC), were employed to create 88 disc-shaped specimens. These comprised 44 specimens fabricated from each material. Each specimen had a diameter of 5 mm and a height of 3 mm. To conduct the experiment, specimens were randomly allocated into four experimental groups, each containing 11 specimens made of each material. This division was accomplished through the use of a stratified random sampling method. The five experimental groups and their respective liquid medications were as follows: Group 1 - montelukast sodium and levocetirizine dihydrochloride syrup, Group 2 - cefixime, Group 3 - sodium valproate, and Group 4 - metronidazole.

To ensure thorough exposure to the medications, all samples underwent a two-minute agitation cycle, which was repeated every 12 h over the course of one week. Following the immersion period, the color stability of all specimens was assessed using a spectrophotometer.

The data obtained from the color stability measurements were subjected to statistical analysis using one-way analysis of variance (ANOVA), followed by a post hoc test. The aim was to determine whether significant differences in color stability were observed among the groups studied.

Results: The mean values and standard deviations of ΔE were calculated. The highest values of ΔE were observed in Group 3 (4.70 ± 1.89), followed by Group 4 (4.04 ± 2.10). Conversely, the lowest ΔE values were observed in Group 2 (3.23 ± 2.02) and Group 1 (3.24 ± 2.31). The calculated p-value was 0.298, and the F-value was 1.269.

Conclusion: This study concludes that both restorative materials, LC composite and LC GIC, are susceptible to discoloration. Sodium valproate exhibited the greatest staining effect on both materials. Conversely, cefixime had the least impact on the color of the LC composite, whereas montelukast had the least effect on the color of LC GIC.

## Introduction

Composite resins have gained significant importance in the field of restorative dentistry. These materials have undergone advancements in their physical and esthetic properties, making them highly sought after for esthetic restorations in dental clinical practice. The increased admiration for composites can be attributed to their ability to provide both functional and esthetic benefits. Additionally, with the decline in the prevalence and severity of dental caries, clinicians are now increasingly focused on conservative and minimally invasive treatment options.

With time, factors such as color stability and surface coarseness have been recognized as crucial for achieving satisfactory outcomes in dental restorations. Moreover, inadequate color matching is a frequent reason for replacing restorations, particularly in the anterior region [1].

The demand for virtually appealing dental outcomes is steadily growing in the field of pediatric dentistry. When it comes to treating the pediatric population, dentists often use various dental materials such as glass ionomers, compomers, and composites. The selection of these materials depends on the specific needs and indications of an individual child [2].

Syrups or suspensions are commonly prescribed for the administration of medications to young children who are experiencing acute and/or chronic illnesses, as they may have difficulties with other forms of medication administration. These liquid formulations typically contain ingredients such as sugars, acids, buffering agents, and approved coloring agents, which can be either oil-based or water-soluble. It is critical to recognize that these medications, which possess a low endogenous pH (potential of hydrogen) and high titratable acidity, can have detrimental effects on dental health. Extended exposure of primary teeth to these liquid formulations can have adverse effects, including tooth surface erosion, intrinsic or extrinsic staining, harm to dental restorations, enamel hardness, and alterations in texture characteristics [3].

Therefore, this study aims to assess the impact of various pediatric drugs on the color stability of two esthetic restorative materials, namely light-cured (LC) composite resin and LC glass ionomer cement (GIC).

## Materials and methods

The present study was conducted in the Department of Pedodontics and Preventive Dentistry at Kalka Dental College, Meerut, with assistance from the Advance Research Lab for various instrumentation.

Specimen preparation

**Figure 1:**
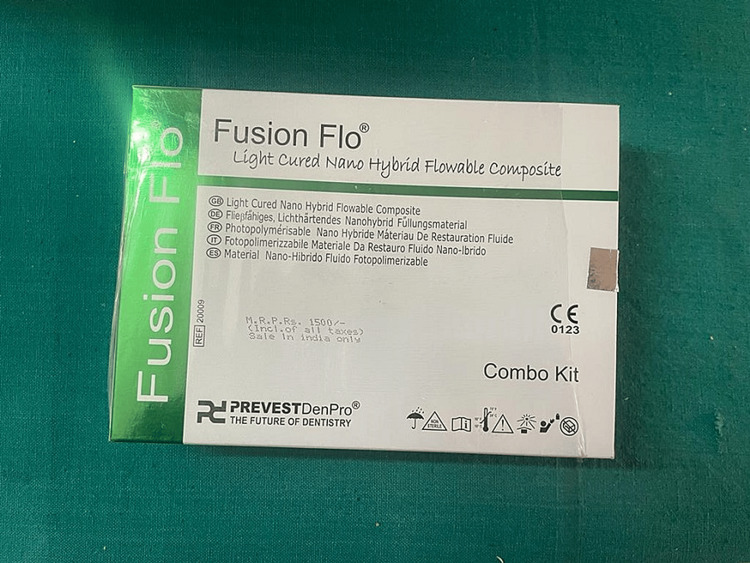
Light-cure composite.

**Figure 2:**
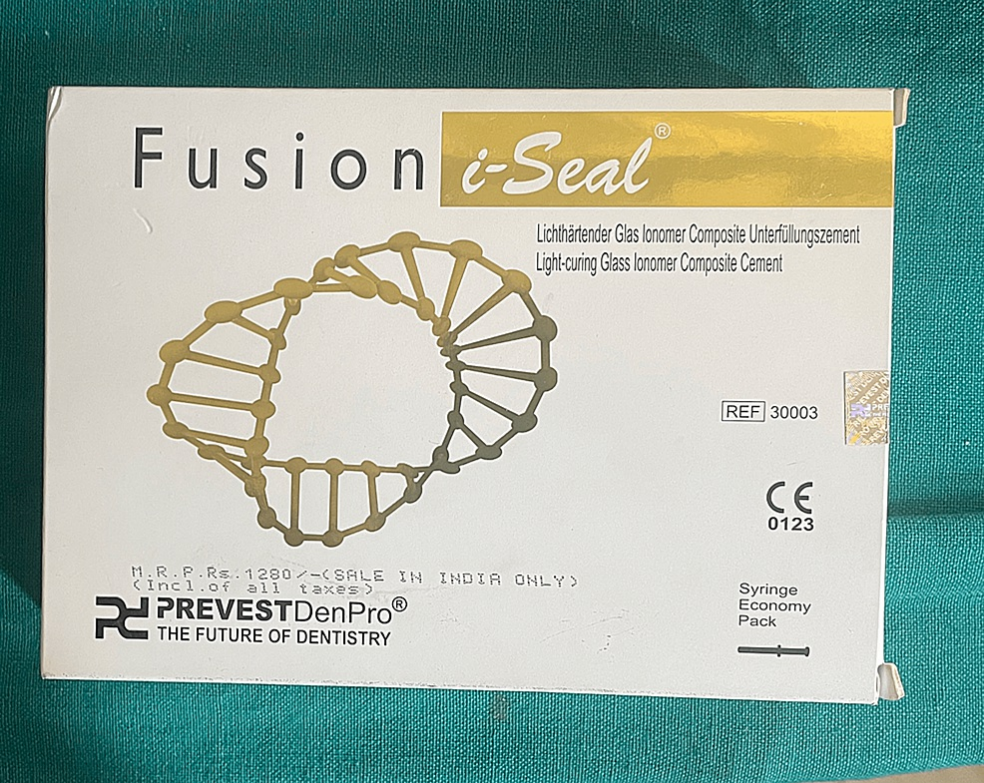
Light-cure GIC. GIC, glass ionomer cement

In this study, two restorative materials were utilized: LC composite resin (Fusion Flo, Prevest USA, as shown in Figure 1) and flowable LC GIC (I-seal, Prevest USA, as shown in Figure 2). Using a silicone mold, a total of 44 cylindrical disc-shaped specimens were created from each material. The dimensions of the specimens were standardized to a height of 3 mm and a diameter of 5 mm.

**Figure 3:**
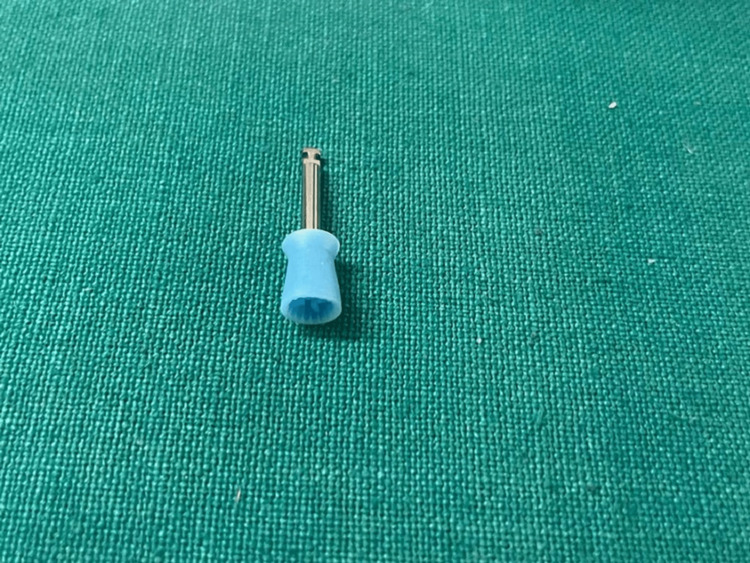
Silicon mold used for preparing samples.

**Figure 4:**
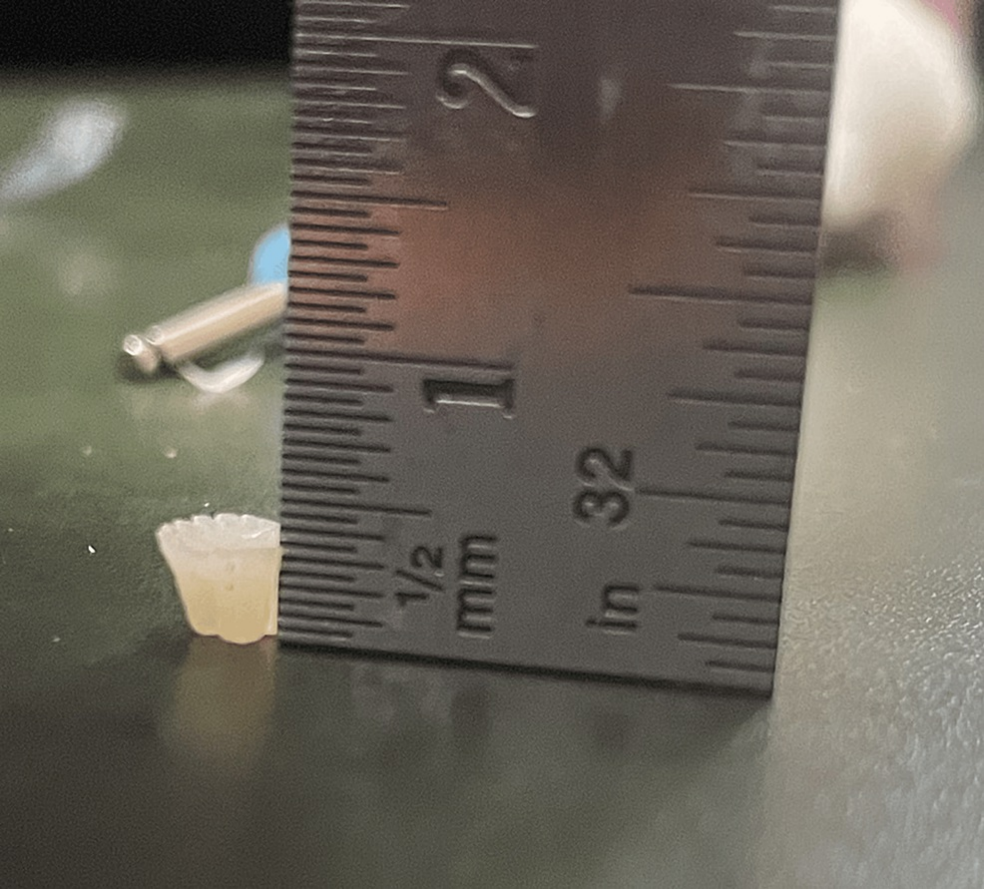
Height of the samples (3 mm).

**Figure 5:**
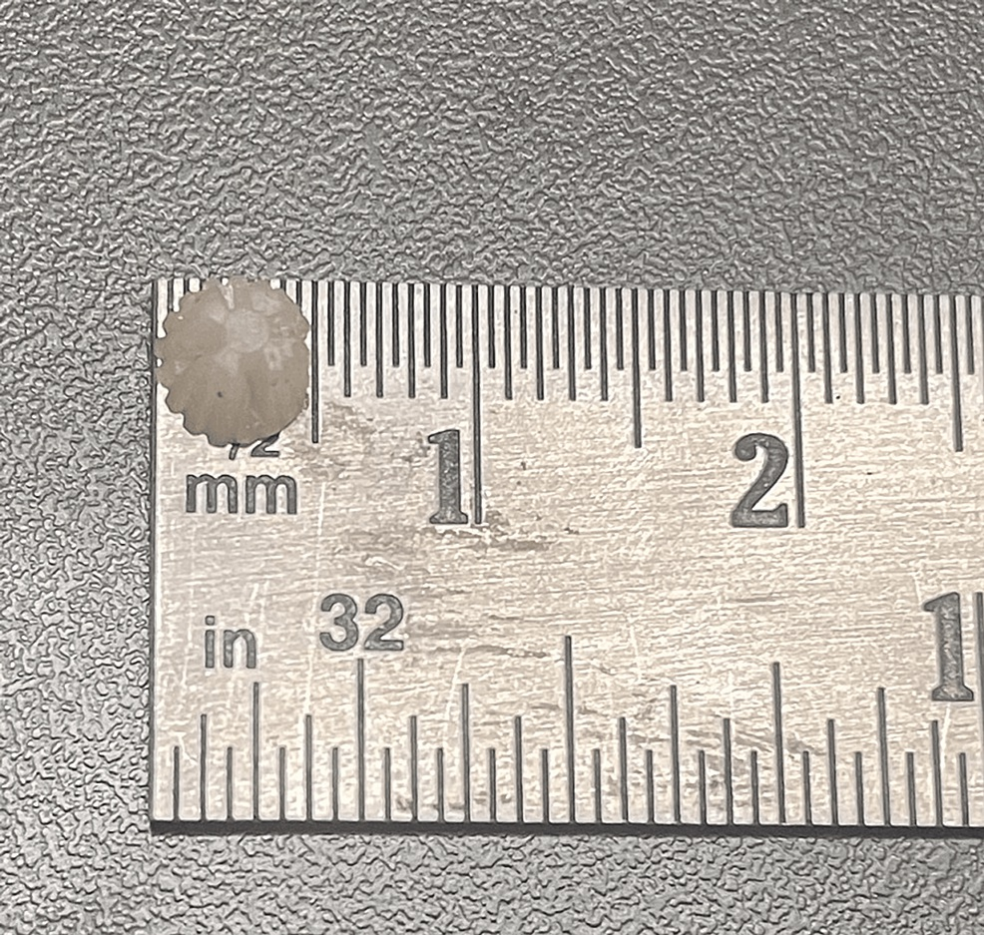
Diameter of the sample (5 mm).

**Figure 6:**
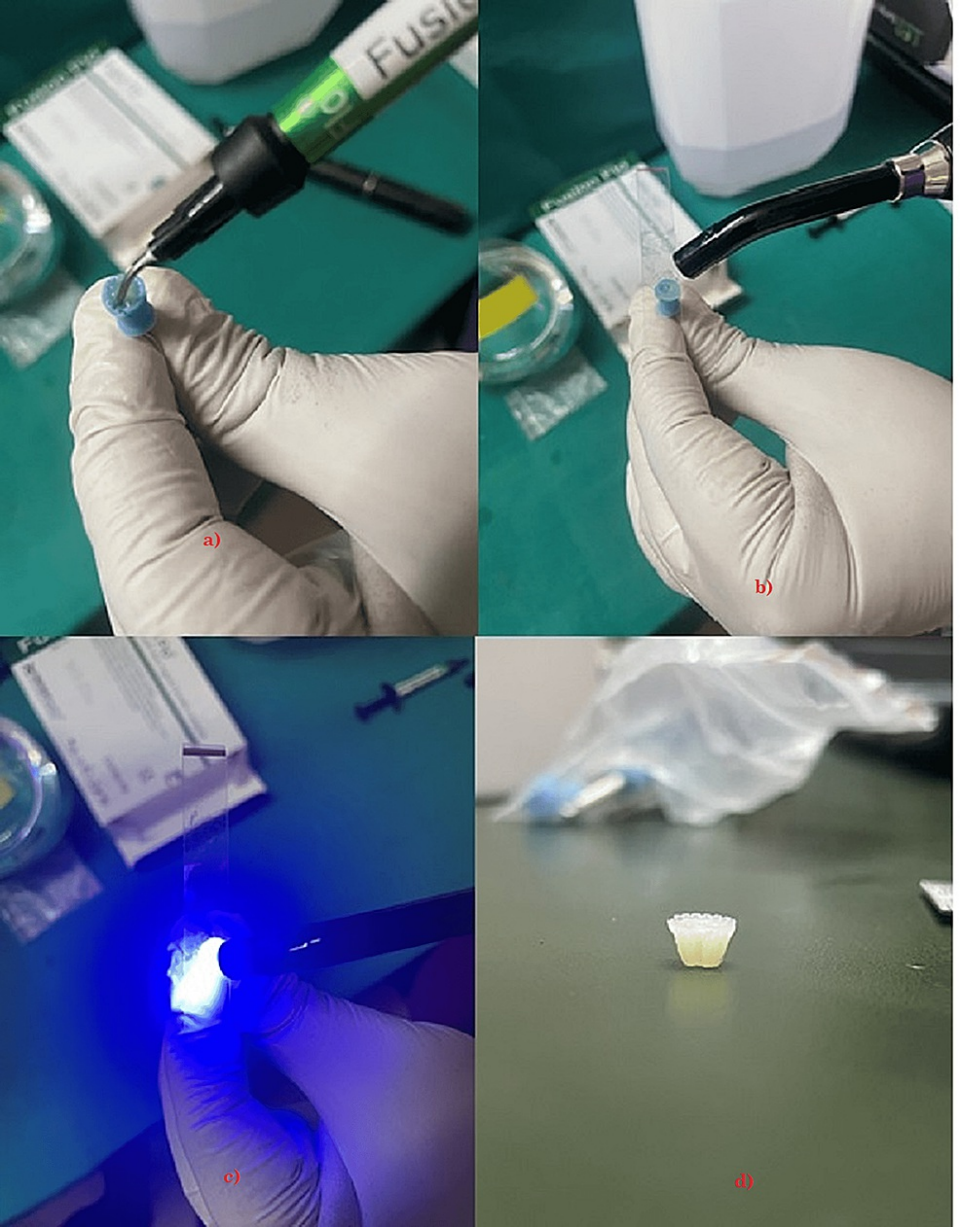
Preparation of the composite samples. a) Showing placing composite in the silicon rubber cup. b) Placing mylar strip over the silicon rubber cup. c) Curing light for 20 s. d) Final shape of samples

To prepare composite specimens, the material was poured into a silicone rubber cup (see Figure 3) and enclosed with a Mylar strip. It was then cured by light for 20 s at a wavelength of 1130 nm using Woodpecker i-Led, as shown in Figure 4. Once the specimens were ready, the size and diameter of each specimen were checked as shown in Figures 5-6.

**Figure 7:**
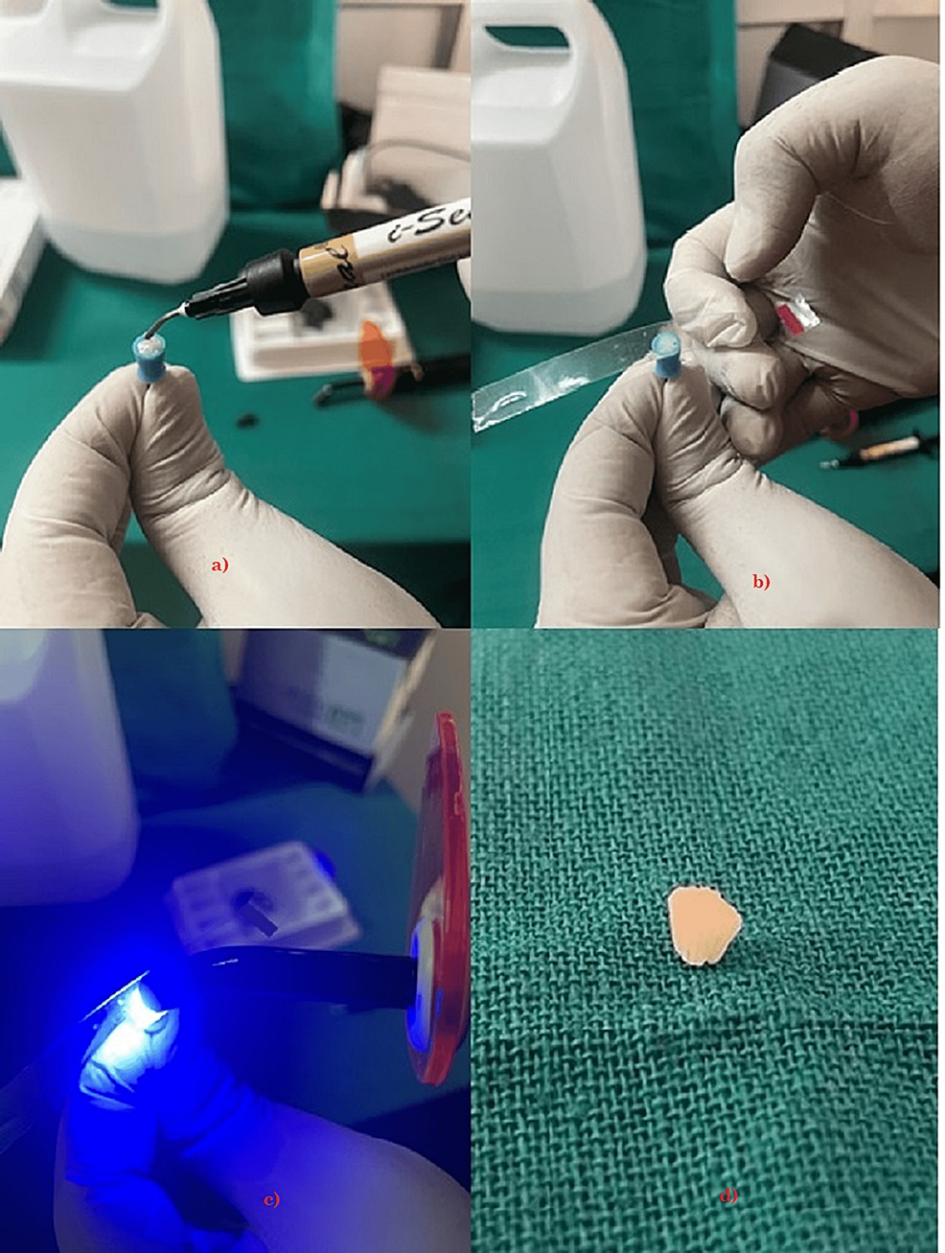
Preparation of GIC samples. a) Showing placing GIC in the silicon rubber cup. b) Placing mylar strip over the silicon rubber cup. c) Curing light for 20 s. d) Final shape of samples GIC, glass ionomer cement

Petroleum jelly was evenly applied to all the surfaces of the silicone rubber cap during the preparation of specimens for LC GIC. The syringe of LC GIC was used directly to place GIC in the rubber silicone cup. Then, the mold is covered with the mylar strip, and the GIC is cured using the LC for 20 s at a wavelength of 1120 nm (refer to Figure 7).

Subgrouping of specimens

From the two restorative materials, a total of 88 specimens were prepared, with 44 specimens from each material. Each group was further divided into four subgroups, each comprising 11 samples. This subdivision was based on the specific pediatric drug formulations that needed to be verified:

Group 1: montelukast sodium and levocetirizine dihydrochloride syrup (Montair LC, Cipla, India)

Group 2: cefixime (Zifi 50, FDC, India)

Group 3: sodium valproate (Valparin, Sanofi, France)

Group 4: metronidazole (Metrogyl; J. B. Chemicals and Pharmaceuticals Ltd., India)

Baseline assessment 

**Figure 8:**
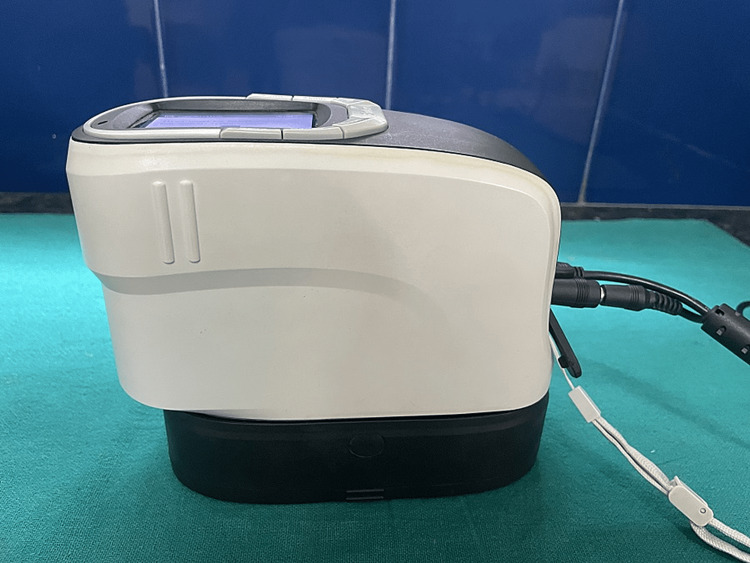
CHNSPES spectrophotometer.

The baseline color values were recorded for each specimen after rinsing it with water and then drying it with autoclaved filter paper. The baseline color values were measured using a spectrophotometer (CHNSPES CS-600A/600B, as shown in Figure 8). The color assessment was conducted using color parameters that corresponded to the average daylight conditions (D65).

Based on the CIELAB (Commission Internationale de l'Eclairage Lab*) color space, the color of the samples was observed. The CIELAB system is a color space that measures chroma and value using three coordinates:

L* represents brightness or lightness (value).

The variables a* and b* are used as numeric indicators for hue and chroma. The a* value denotes the red/green axis, while the b* value represents the yellow/blue axis.

The color difference (ΔL*) between two objects can be determined using the CIELAB system. The study employed a specific formula provided in the study to calculate the ΔL* value:

ΔE (L * a * b*) = ([ΔL*]2 + [Δa*]2 + [Δb*]2 ) ½ [4].

pH cycling

To simulate oral conditions, all specimens underwent pH cycling during the study. This process involved immersing the specimens in a demineralizing solution for 12 h each day, followed by immersion in a remineralizing solution for another 12 h for 1 week. [R1] The pH cycling mimicked the natural cycle of demineralization and remineralization that occurs in the oral cavity. 

**Figure 9:**
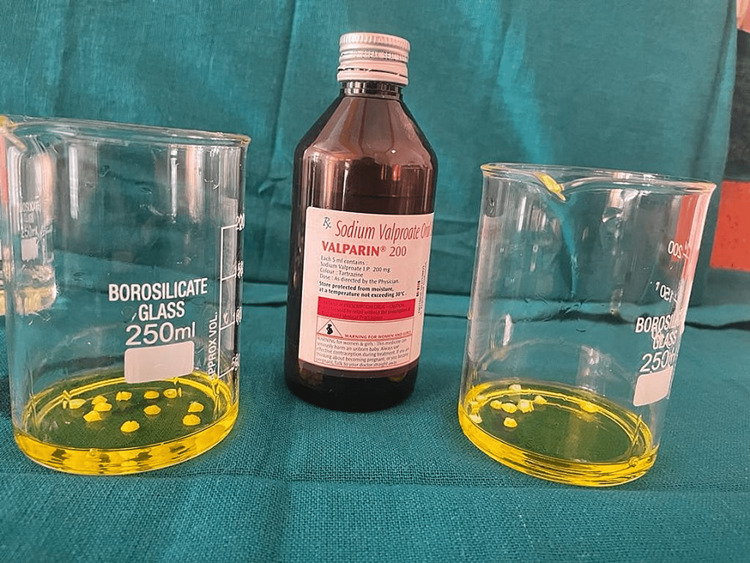
Samples were dipped in the pediatric drugs for 2 min, twice a day.

**Figure 10:**
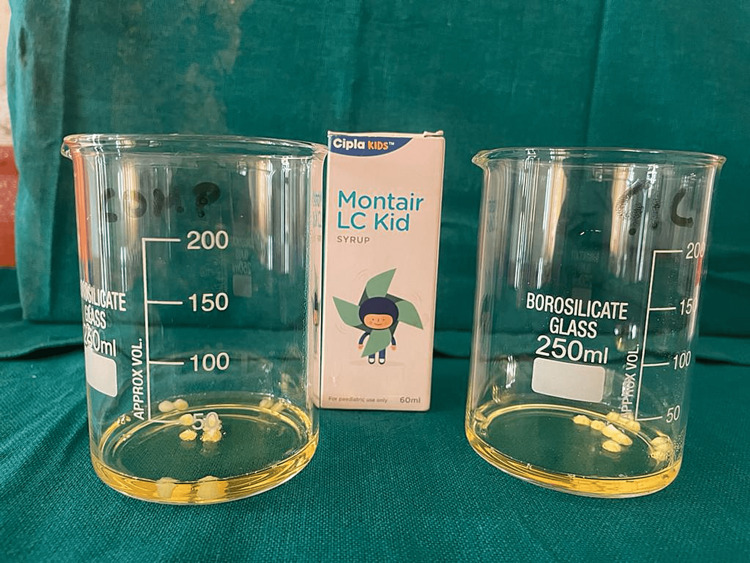
Samples were dipped in the pediatric drugs for 2 min, twice a day.

**Figure 11:**
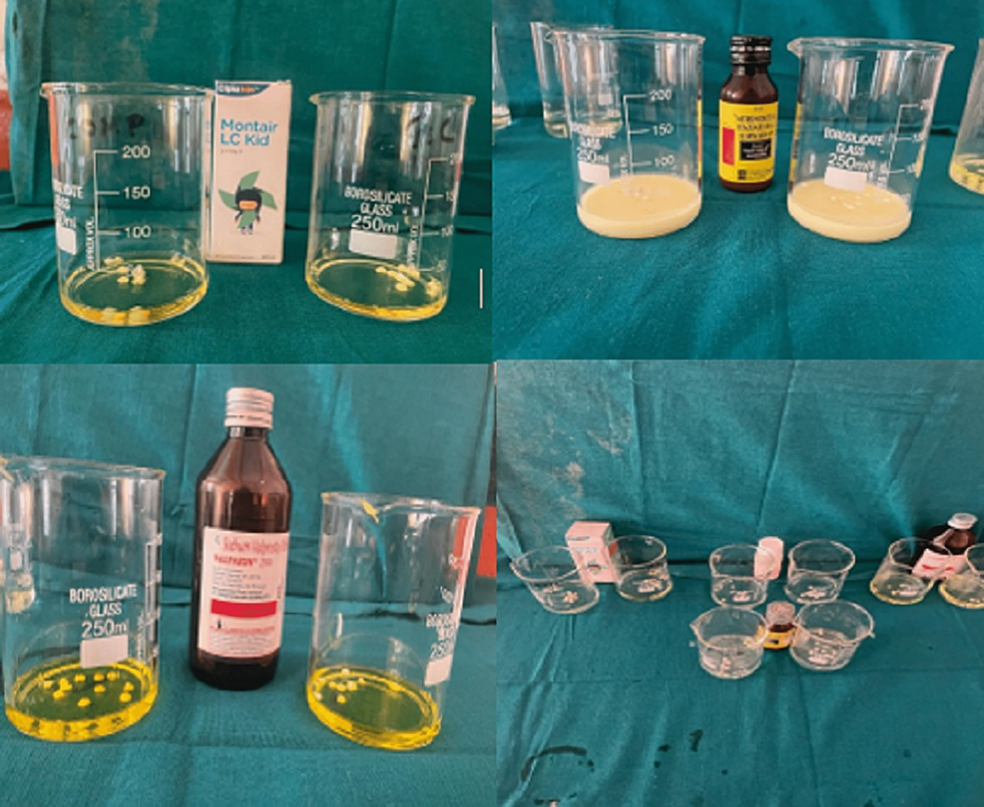
All the samples immersed in the solution.

The samples were immersed in a demineralizing solution for 12 h. Next, the samples were placed in a beaker and submerged in 15 mL of a pediatric drug for 2 min as shown in Figures 9-10. Then, the samples were placed in a remineralizing solution for 12 h. Samples were then once again immersed in the drug for 2 min as shown in Figure 11. This cycle was repeated for one week.

Production of remineralization and demineralization solutions

**Figure 12:**
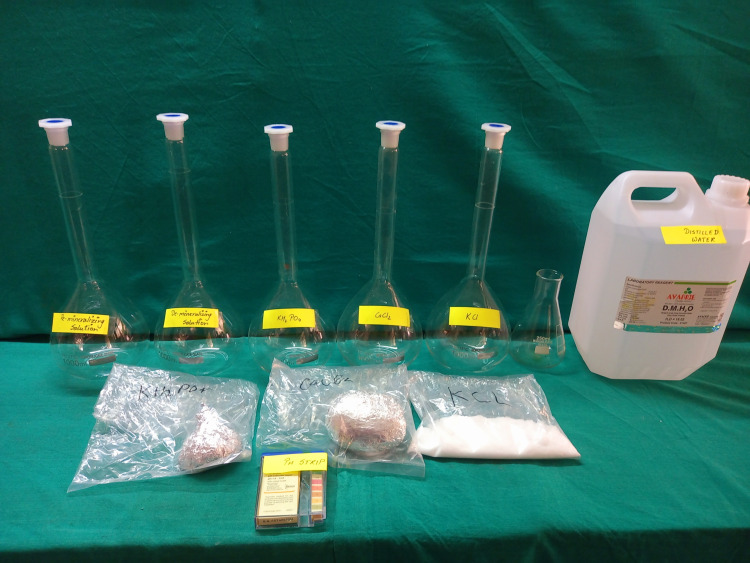
The material used for preparation of demineralizing solution and demineralizing solution.

**Figure 13:**
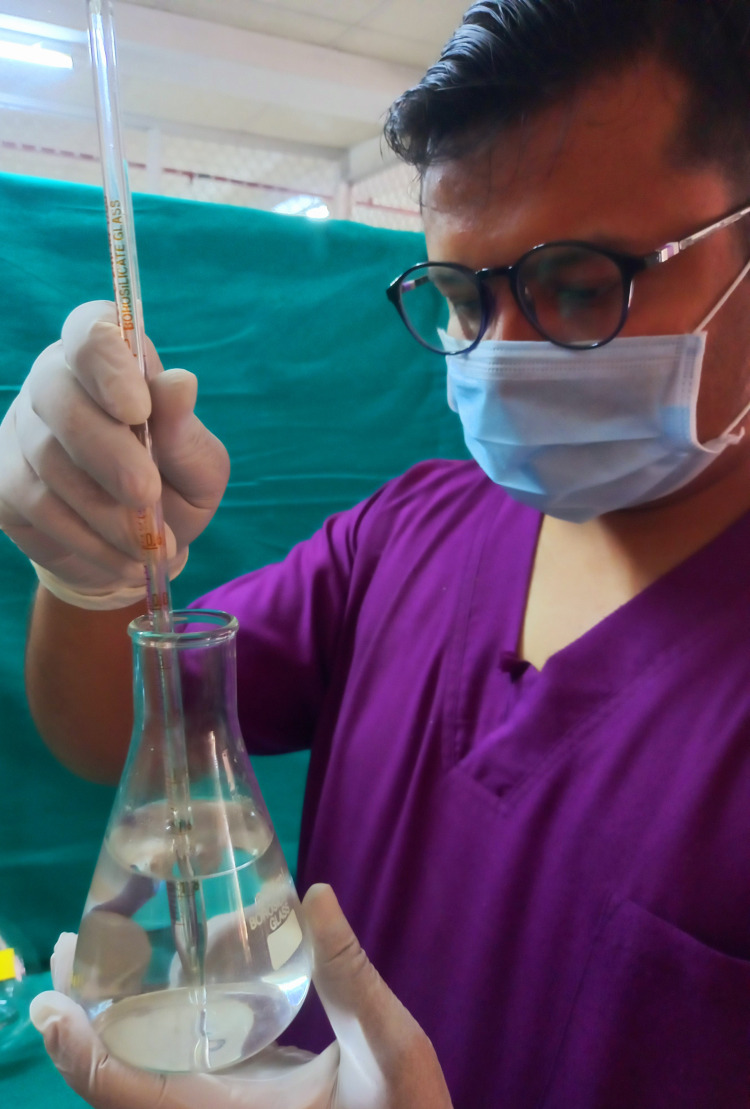
Preparing the solutions.

The buffered de-mineralizing and re-mineralizing solutions were prepared using high-quality chemicals of analytical grade and de-ionized water as shown in Figures 12-13. The demineralizing solution was formulated with 1.5 mmol of calcium chloride, 0.9 mmol of potassium phosphate, and 50 mmol of acetic acid. The pH (potential of hydrogen) of the demineralizing solution was adjusted to 5 using a 1.00 mol sodium hydroxide solution. The remineralizing solution, on the other hand, consisted of 1.50 mmol of calcium chloride, 0.9 mmol of sodium phosphate, and 0.15 mol of potassium chloride, with a pH of 7. These solutions were carefully prepared to simulate the conditions found in the oral environment.

Statistical analysis

The data for this study were entered into Microsoft Excel 2007 and analyzed using SPSS software version 23.0 (IBM Inc., Armonk, NY). Descriptive statistics were used to calculate the arithmetic mean and standard deviation. The significance level for this study was set at 5%. To compare the mean scores between groups, intergroup comparisons were conducted using either the one-way analysis of variance (ANOVA) or the Kruskal-Wallis test, depending on the normality of the data.

## Results

The calculations of the means and standard deviations of ΔE were performed. The one-way ANOVA test was conducted on the resin group, as shown in Table 1, revealing a significance level of 0298. In the resin group (see Table 2), the maximum ΔE value was observed in Group 3 (4.70 ± 1.89), followed by Group 4 (4.04 ± 2.10).

**Table 1:** One-way analysis of variance.

	Sum of squares	df	Mean square	F	Sig.
Between groups	16.684	3	5.561	1.269	0.298 (NS)
Within groups	175.245	40	4.381		
Total	191.928	43			

**Table 2:** Intergroup comparison between four drug formulations in composite.

Mean		Standard deviation	Standard error	Minimum	Maximum
I	3.2445	2.32261	0.70029	0.90	6.85
II	3.2338	2.02999	0.61206	0.43	6.08
III	4.7092	1.89608	0.57169	0.74	7.77
IV	4.0473	2.10095	0.63346	0.37	7.32

The least value of ΔE was observed in Group 2 (3.23 ± 2.02) and Group 1 (3.24 ± 2.31), with p = 0.298 and F = 1.269. The post-hoc analysis in Table 3 revealed that there is a non-significant difference between all four of these groups.

**Table 3:** Intergroup comparison analysis (post-hoc tukey test). NS, not significant

Intergroup comparison	Mean difference	Standard error	Sig.	
				I vs. II	0.01061	0.89251	0.991 NS	
I vs. III	-1.46476	0.89251	0.109 NS	
I vs. IV	-0.80283	0.89251	0.374 NS	
II vs. III	-1.47538	0.89251	0.106 NS	
II vs. IV	-0.81344	0.89251	0.368 NS	
III vs. IV	0.66193	0.89251	0.463 NS	

In the GIC, the intergroup comparison shown in Table 4 was conducted to determine the material with the highest staining among all the groups provided. The one-way ANOVA test results shown in Table 5 indicate that the significance in the GIC group was 0.00. In Group 3, the highest ΔE value was observed (3.61 ± 2.27), followed by Group 4 (3.36 ± 2.22).

**Table 4:** Intergroup comparison between four drug formulations in GIC. GIC, glass ionomer cement

Mean		Standard deviation	Standard error	Minimum	Maximum
I	0.5576	0.36372	0.10967	0.14	1.30
II	2.0393	0.65847	0.19854	1.05	3.25
III	3.6123	2.27040	0.68455	0.71	7.14
IV	3.3684	2.22045	0.66949	1.07	7.65

**Table 5:** One-way analysis of variance.

	Sum of squares	df	Mean square	F	Sig.
Between groups	65.251	3	21.750	8.168	0.000
Within groups	106.510	40	2.663		
Total	171.761	43			

The minimum value of ΔE was observed in Group 1 (0.55 ± 0.36), with p = 0.001 and F = 8.168. The post-hoc analysis in Table 6 shows a significant difference between Groups I and II, Groups I and III, Groups I and IV, Groups II and III, and Groups II and IV. The results indicate no significance between Groups III and IV. 

**Table 6:** Intergroup comparison analysis (post-hoc tukey test). S, significant; NS, not significant

Intergroup comparison	Mean difference	Standard error	Sig.	
				I vs. II	-1.48174^*^	0.69580	0.039 (S)	
I vs. III	-3.05474^*^	0.69580	0.001 (S)	
I vs. IV	-2.81078^*^	0.69580	0.001 (S)	
II vs. III	-1.57300^*^	0.69580	0.029 (S)	
II vs. IV	-1.32904	0.69580	0.043 (S)	
III vs. IV	0.24396	0.69580	0.728 (NS)	

## Discussion

The primary objective was to assess the impact of four distinct pediatric drug formulations on the color stability of LC composite resin and LC GIC. There is a dearth of existing research on the color stability of LC GIC, which further highlights the imperativeness of conducting this study.

In order to minimize subjective perceptions and potential bias related to human color sensitivity, we utilized a spectrophotometer for color analysis. This approach offered benefits such as sensitivity, repeatability, and objectivity in our measurements.

Our findings indicate that the group treated with composite resin and exposed to sodium valproate exhibited the most pronounced color changes, while the group treated with GIC and exposed to metronidazole demonstrated the least color alteration. Furthermore, significant color changes were observed in the GIC group compared to the LC composite resin group for all four drug formulations.

A study conducted in 2019 investigated three different restorative materials: composite resin, zirconomer improved, and GIC. They also explored the effects of five pediatric drug formulations. Their study concluded that GIC demonstrated superior color stability across all drug formulations. The composite resin group exhibited the highest degree of color alteration, while GIC demonstrated color stability that was lower than GIC but higher than composite resin [5].

In a separate study conducted in 2017, the researchers investigated the properties of commonly used pediatric drugs on light-polymerized specimens, including compomer, composite, and GIC. The findings revealed that composites experienced notable discoloration when exposed to pediatric drugs, while GIC exhibited greater resistance to the discoloration effects of these drug formulations [2].

The discoloration observed in the composite resin can be attributed to several factors, such as water absorption. This absorption may weaken the adhesive bond between the resin matrix and filler particles, leading to the formation of microcracks or interfacial gaps. Additionally, the presence of an initiator-activator system and water absorption by the monomers in composites may enhance the penetration of stains, ultimately resulting in discoloration [4].

The study conducted in 1988 proposed that the silanization process of filler particles discarded in resin-based composites plays a role in discoloration. This is due to the hydrophilic nature of silane, which can contribute to increased water absorption. Consequently, due to a higher proportion of silane, such as Tetric-N Ceram, in the composite resins, they tend to exhibit higher staining values. Based on this information, it can be inferred that composite resins with a higher content of resin matrix, a higher concentration of silane, and larger-sized filler particles are more prone to discoloration [6].

Limitations of the study

Less number of articles were used to support the hypothesis. The time for immersion of restoration into drugs could be increased to observe more effective results. It is important to consider that drug actions may differ in vitro and in vivo.

## Conclusions

This study concludes that both the LC composite and LC GIC are susceptible to staining, with the drug Valparin having the most noticeable effect on discoloration for both materials. On the other hand, cefixime had the least impact on the color stability of the LC composite, while montelukast had the least effect on the color of the LC GIC. The potential for staining of pediatric drug formulations when combined with various restorative materials, such as composite resin with metronidazole and sodium valproate, is an important factor to consider. It is imperative to share this information with pediatricians, pediatric dentists, and parents in order to raise awareness about the potential risk of tooth surface and restoration discoloration. Therefore, it is vital to have knowledge about which restorative material to use in order to minimize the risk of discoloration and ensure optimal holistic outcomes.
